# Investigating visual field measurements on perimetry and ganglion cell complex on optical coherence tomography in patients with suprasellar tumours

**DOI:** 10.4102/jcmsa.v4i1.380

**Published:** 2026-08-31

**Authors:** Saajidah Gaibie, Susan Williams

**Affiliations:** 1 Department of Neurosciences, Faculty of Health Sciences, University of the Witwatersrand, Johannesburg, South Africa

**Keywords:** suprasellar tumours, chiasmal compression, optical coherence tomography, ganglion cell complex, visual fields, perimetry, structure–function relationship

## Abstract

**Background:**

Suprasellar tumours can cause compressive optic neuropathy with preventable visual loss. Standard automated perimetry is central to assessment but is subjective; optical coherence tomography (OCT) provides ganglion cell complex (GCC) metrics, yet structure–function relationships in chiasmal compression remain unclear. This study evaluated the relationship between GCC thickness and visual field (VF) loss (10-2 and 30-2).

**Methods:**

Prospective observational study of patients with suprasellar tumours undergoing OCT GCC imaging and Humphrey VF testing (10-2 and 30-2). Ganglion cell complex abnormality was defined as a machine-flagged red value. Visual field abnormality was defined on the pattern deviation (PD) map as a scotoma (≥ 3 adjacent points with *p* < 5%). Associations between GCC thickness and VF PD were assessed, and agreement between binary classifications was evaluated.

**Results:**

Seventeen eyes were included (mean age 50.7 years; 70.0% female). Visual field abnormality was frequent (10-2: 82.4%; 30-2: 94.1%), while GCC was abnormal in 58.8%. Mean PD was −6.84 dB (10-2) and −8.66 dB (30-2). Ganglion cell complex thickness correlated with PD at 10-2 (*p* < 0.001) and 30-2 (*p* = 0.001). Agreement was moderate for GCC versus VF 10-2 (κ = 0.469) and slight for GCC versus VF 30-2 (κ = 0.164).

**Conclusion:**

Visual field abnormalities were common, with 30-2 classifying more eyes as abnormal despite normal GCC. Ganglion cell complex thickness showed a graded association with VF loss on both strategies, supporting OCT GCC as an adjunct for quantifying chiasmal compression–related dysfunction; however, binary OCT classification may miss milder functional deficits.

**Contribution:**

Supports combined OCT–perimetry interpretation and strategy-aware follow-up.

## Introduction

Suprasellar tumours are space-occupying lesions originating from the pituitary gland or adjacent structures. They remain a significant cause of potentially preventable visual impairment. Visual dysfunction generally results from compression of the optic nerves and optic chiasm. This leads to compressive optic neuropathy, characterised by functional impairments on perimetry and structural damage to retinal ganglion cell axons and cell bodies.^1^ Vision loss affects independence, employability and quality of life, but it may be partially reversible with timely treatment.^1^ Early detection and precise monitoring of visual involvement are therefore of paramount clinical importance. In the South African public-sector setting, patients may present with established optic pathway compromise, highlighting the need for practical tools that quantify structure–function impairment and support monitoring.

Standard automated perimetry remains the clinical cornerstone for assessing compressive optic neuropathy because it quantifies visual field (VF) sensitivity and supports localisation along the visual pathway.^2^ Although bitemporal hemianopia is classically described as the hallmark pattern of pituitary-region disease, VF loss in suprasellar tumours is heterogeneous and depends on tumour size, configuration, and its relationship to the optic nerves, chiasm and optic tracts.^3,4^ Perimetry is also inherently subjective and depends on patient cooperation, attention and adequate visual acuity (VA). Reliability may be reduced by fatigue and test duration, which can complicate monitoring in routine clinical practice, particularly in busy clinics and among patients with more advanced disease or limited test reliability.^2^ The choice of perimetric strategy may also affect the detection of functional loss in the macula. The standard 30-2 testing encompasses a broader VF but offers lower spatial resolution within the central 10 degrees. Conversely, 10-2 testing provides a higher-resolution evaluation of the central VF, which may more accurately reflect the integrity of macular ganglion cells. Evidence from central field examinations in chiasmal compression cases underscores the clinical significance of central testing in correlating functional loss with optical coherence tomography (OCT) parameters.^5^ The choice of programme in pituitary-region pathology may also affect detection efficacy, thereby emphasising the necessity for strategy-conscious interpretation during monitoring patients.^6^

In parallel, advances in OCT have facilitated the rapid and objective quantification of structural damage within the optic nerve and macula. Optical coherence tomography produces high-resolution cross-sectional images of the retina, enabling precise measurement of the retinal nerve fibre layer and macular ganglion cell complex (GCC). In cases involving suprasellar tumours, thinning of the retinal nerve fibre layer and GCC has been documented even in some patients lacking overt chiasmal compression, thereby supporting the potential utility of OCT in identifying early or subclinical damage.^7^ In instances of established chiasmal compression, loss of GCC has been demonstrated in tumour-related compression, further endorsing its role as a structural marker for compressive optic neuropathy.^8^ Macular GCC asymmetry has also been proposed as a sensitive indicator of chiasmal involvement, possibly preceding detectable VF defects.^9^ Moreover, OCT GCC analysis has been reported to identify compressive chiasmopathy in patients with normal VF, thus reinforcing its prospective value as a complementary diagnostic modality in select clinical scenarios.^10^

Despite this promise, translating OCT findings into clinically intuitive predictions of functional loss remains challenging. Structure–function relationships are well described in glaucoma, but fewer studies have characterised these relationships in compressive optic neuropathy due to suprasellar masses.^11,12^ As a result, it remains uncertain how reliably macular GCC abnormalities correspond to VF loss, and whether OCT metrics can meaningfully complement perimetry when perimetry is unreliable, not feasible or when early detection is prioritised. This gap is clinically relevant because establishing predictable structure–function relationships could strengthen early detection, improve monitoring and support more efficient testing strategies in settings where clinic time and patient reliability are constrained.^12,13^

**Aim:** To evaluate the relationship between structural parameters on OCT (macular GCC measurements) and functional parameters on standard automated perimetry (30-2 and 10-2 VF) in patients with suprasellar tumours.

**Objectives:** (1) To describe the frequency of abnormalities on 30-2 and 10-2 VF testing in the study population; (2) to describe macular GCC abnormalities on OCT; (3) to assess agreement between GCC abnormality and VF abnormality; and; (4) to examine quadrant-level structure–function relationships between GCC thickness and VF measures.

## Research methods and design

### Study design

This was a prospective, observational, cross-sectional quantitative study conducted to evaluate structure–function relationships in patients with suprasellar tumours referred for neuro-ophthalmic assessment. The primary analytic focus was the relationship between macular GCC thickness, measured by OCT, and functional performance on standard automated perimetry using the 30-2 and 10-2 VF strategies. Secondary analyses assessed agreement between binary classifications of abnormal structure and abnormal function.

### Setting

The study was conducted at Charlotte Maxeke Johannesburg Academic Hospital, a tertiary academic referral centre located in Johannesburg, Gauteng, South Africa. Participants were assessed in the ophthalmology clinic following referral for evaluation of VF defects associated with suprasellar masses. Recruitment took place from 01 January 2025 to 31 May 2025.

### Study population and sampling strategy

The source population comprised adult patients with suprasellar tumours who were referred for assessment of suspected or confirmed VF abnormalities. Convenience sampling was employed, encompassing all eligible patients referred during the study period.

Participants met the inclusion criteria if they were aged 18 years or older, provided written informed consent, possessed adequate VA for reliable perimetry (VA ≥ 6/24), and yielded reliable VF indices, as indicated by fixation losses, false-positive errors and false-negative errors each being less than 15%. Patients were excluded if they had ocular comorbidities that could cause perimetric defects or confound OCT or VF interpretation, including glaucoma, maculopathy, degenerative myopia, visually significant cataract or corneal opacities. Additionally, participants were excluded if reliable VF testing could not be obtained on two separate attempts.

### Data collection

All eligible participants underwent a comprehensive ophthalmic assessment to confirm eligibility and characterise ocular status. Visual acuity was recorded, followed by slit-lamp biomicroscopy and fundus examination performed by the principal investigator. Standard automated perimetry was performed on the Humphrey Field Analyser using both 30-2 and 10-2 strategies, and macular GCC measurements were obtained using OCT (ZEISS Cirrus 4000). Perimetry and OCT acquisition were performed by clinic optometrists, and all study variables were recorded using a structured data collection tool.

**FIGURE 1:**
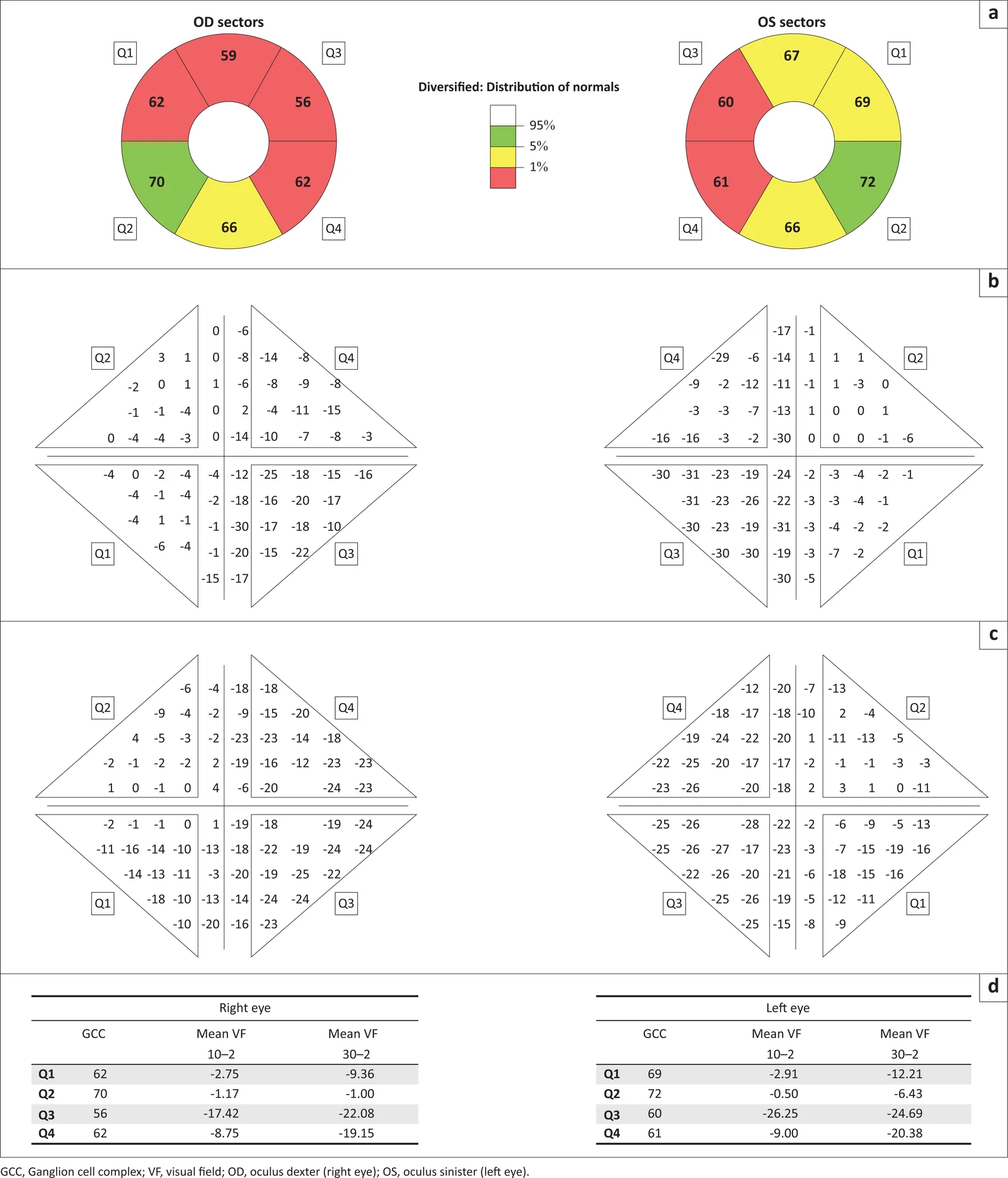
Ganglion cell complex-visual field sector mapping. Patient 1: (a) Ganglion cell complex printout in both eyes; (b) Humphrey automated perimetry 10-2 pattern deviation printout in both eyes; (c) and 30-2 pattern deviation outputs in both eyes labelled according to the correlating quadrants (Q1-4); (d) mean values from each perimetry quadrant correlated with the corresponding quadrant on ganglion cell complex.

Structural variables included mean macular GCC thickness and software-derived quadrant GCC values, used for quadrant-level descriptive summaries and sectoral structure–function analyses (Table 1 and Figure 1). For sectoral structure–function mapping, quadrants were labelled according to the corresponding VF–GCC relationship shown in Table 1: Quadrant 1 = VF inferior-nasal/GCC superior-temporal; Quadrant 2 = VF superior-nasal/GCC inferior-temporal; Quadrant 3 = VF inferior-temporal/GCC superior-nasal; and Quadrant 4 = VF superior-temporal/GCC inferior-nasal. For binary classification, GCC abnormality was defined as a machine-flagged red value on the OCT normative comparison display. Functional variables were derived from VF pattern deviation (PD) outputs. For quadrant-level VF analyses, the VF test grid was divided into four quadrants using the horizontal and vertical meridians and numbered according to the corresponding sectors on the GCC, as outlined in Table 1. For each VF quadrant, the pointwise PD values within that quadrant were averaged to derive a mean quadrant PD value. This was performed separately for the 10-2 and 30-2 Humphrey VF strategies. Further descriptions and visual explanations are illustrated in Figure 1. Visual field abnormality was defined on the PD probability map as a scotoma, defined as a cluster of three or more adjacent points with *p* < 5%. Visual field reliability indices were reviewed for each test; tests not meeting predefined thresholds were repeated where feasible, and data were excluded if reliability criteria were not met.

**TABLE 1:** Quadrant definitions with correlating visual field and ganglion cell complex.

Quadrants	VF	GCC
Quadrant 1	Inferior-nasal	Superior-temporal
Quadrant 2	Superior-nasal	Inferior-temporal
Quadrant 3	Inferior-temporal	Superior-nasal
Quadrant 4	Superior-temporal	Inferior-nasal

GCC, ganglion cell complex; VF, visual field.

### Data analysis

Data were recorded using Microsoft Excel and subsequently analysed with Stata (StataCorp, College Station, TX, United States). Continuous variables were summarised as means with standard deviations or medians with interquartile ranges (IQR), depending on distribution. Categorical variables were presented as counts and percentages. Analyses were performed at the eye level.

The agreement between binary classifications, namely abnormal or normal OCT GCC status and abnormal or normal VF status on 10-2 and 30-2 testing, was evaluated, with observed agreement documented. Structure–function relationships were analysed between mean GCC thickness and mean PD on 10-2 and 30-2 assessments. Quadrant-level structure–function analyses were performed using the mean VF PD value derived for each quadrant and the corresponding mapped GCC quadrant value. Differences in GCC thickness and VF indices were compared between GCC-abnormal and GCC-normal groups. Snellen VA was converted to logarithm of the minimum angle of resolution (logMAR) for analytical purposes and categorised according to World Health Organization (WHO) guidelines: no visual impairment, defined as logMAR ≤ 0.30, equivalent to ≥ 6/12; mild visual impairment, defined as logMAR > 0.30 to 0.48, equivalent to < 6/12 to 6/18; and moderate visual impairment, defined as logMAR > 0.48 to 1.00, equivalent to < 6/18 to 6/60. When applicable, associations between VA categories and binary outcomes were examined, particularly when expected cell counts were small. Missing data were addressed through complete-case analysis in each comparison. Statistical significance was established at a two-sided *p*-value < 0.05.

### Ethical considerations

Ethical clearance to conduct this study was obtained from the University of the Witwatersrand Human Research Ethics Committee (Medical) (No. M240926). Participation was voluntary, and written informed consent was obtained from all participants prior to enrolment. Confidentiality was maintained by excluding names, hospital numbers and other direct identifiers from the analytical dataset. Paper-based documents were stored securely, and electronic data were stored on a password-protected computer with access restricted to the study team; study files were additionally password-protected.

## Results

### Participant flow and baseline characteristics

Eighteen patients (36 eyes) were assessed for eligibility. At the patient level, 10 patients met the study eligibility requirements; however, inclusion was determined at the eye level. Of the 20 eyes from these 10 eligible patients, three did not meet the eye-level inclusion criteria and were excluded, leaving 17 eyes for the final analysis. Eight patients (16 eyes) were excluded during screening, predominantly because VA was below the minimum threshold necessary for reliable testing. In two patients, VF testing remained unreliable on two separate occasions.

The cohort comprised 10 patients, with a mean age of 50.7 ± 17.2 years (range, 25–70). Seven patients were female (70.0%), and three were male (30.0%). Macroadenoma was the primary diagnosis in 80.0% of the patients, whereas 20.0% presented with macroadenoma accompanied by apoplexy.

### Eye-level structure and function

Seventeen eyes were analysed. Mean GCC thickness, calculated as the average across four GCC quadrants per eye, was 69.26 μm ± 17.10 μm, with a median of 66.0 μm and an IQR of 56.20 μm – 80.50 μm. The mean PD was −6.84 dB ± 5.76 dB on 10-2 testing and −8.66 dB ± 7.66 dB on 30-2 testing (refer to Table 2).

**TABLE 2:** Eye-level summary.

Patient	Eye	Mean GCC (μm)	Mean PD 10-2 (dB)	Mean PD 30-2 (dB)	VF 10-2	VF 30-2	GCC status
1	Right	62.5	−7.52	−12.90	*Abnormal*	*Abnormal*	*Abnormal*
	Left	65.5	−9.66	−15.93	*Abnormal*	*Abnormal*	*Abnormal*
2	Right	81.8	−1.50	−5.02	**Normal**	*Abnormal*	**Normal**
	Left	79.8	−1.66	−2.34	**Normal**	*Abnormal*	**Normal**
3	Right	90.0	−4.49	−1.70	*Abnormal*	*Abnormal*	**Normal**
	Left	99.8	−1.25	−1.95	**Normal**	**Normal**	**Normal**
4	Right	53.8	−16.92	−3.37	*Abnormal*	*Abnormal*	*Abnormal*
	Left	66.0	−2.21	−3.26	*Abnormal*	*Abnormal*	*Abnormal*
5	Left	46.2	−13.36	−26.34	*Abnormal*	*Abnormal*	*Abnormal*
6	Right	94.2	−6.44	−9.24	*Abnormal*	*Abnormal*	**Normal**
7	Right	78.8	−1.88	−2.78	*Abnormal*	*Abnormal*	**Normal**
	Left	80.5	−2.81	−1.86	*Abnormal*	*Abnormal*	**Normal**
8	Right	48.8	−15.79	−17.38	*Abnormal*	*Abnormal*	*Abnormal*
	Left	44.0	−17.29	−19.04	*Abnormal*	*Abnormal*	*Abnormal*
9	Right	57.8	−3.40	−4.74	*Abnormal*	*Abnormal*	*Abnormal*
	Left	71.8	−2.21	−3.85	*Abnormal*	*Abnormal*	*Abnormal*
10	Right	56.2	−7.84	−15.53	*Abnormal*	*Abnormal*	*Abnormal*

Note: Values shown are eye-level means calculated across four quadrants. The bold indicates normal results and the italics indicates abnormal results, for emphasis.

dB, decibel; GCC, ganglion cell complex; PD, pattern deviation; VF, visual field.

Utilising predefined binary classifications, VF 10-2 was identified as abnormal in 82.4% of the eyes, while VF 30-2 was deemed abnormal in 94.1%. Ganglion cell complex was considered abnormal in 58.8% of cases (see Table 2). Two eyes were normal on VF 10-2 but showed abnormalities on VF 30-2 (both eyes from patient 2). One eye remained normal across both VF modalities (Patient 3, left eye).

### Differences by ganglion cell complex classification

The median GCC thickness was 57.0 μm (IQR 50.05 to 64.75) in eyes with abnormal GCC, compared to 81.8 μm (IQR 80.15 to 92.10) in eyes with normal GCC (*p* < 0.001). The median PD on the 10-2 test was −8.75 dB (IQR −15.18 to −4.43) in eyes with abnormal GCC, versus −1.88 dB (IQR −3.65 to −1.58) in eyes with normal GCC (*p* = 0.010). The median PD on the 30-2 test was −14.21 dB (IQR −17.02 to −4.07) in eyes with abnormal GCC, compared to −2.34 dB (IQR −3.90 to −1.91) in eyes with normal GCC (*p* = 0.007).

### Structure–function relationships

**FIGURE 2:**
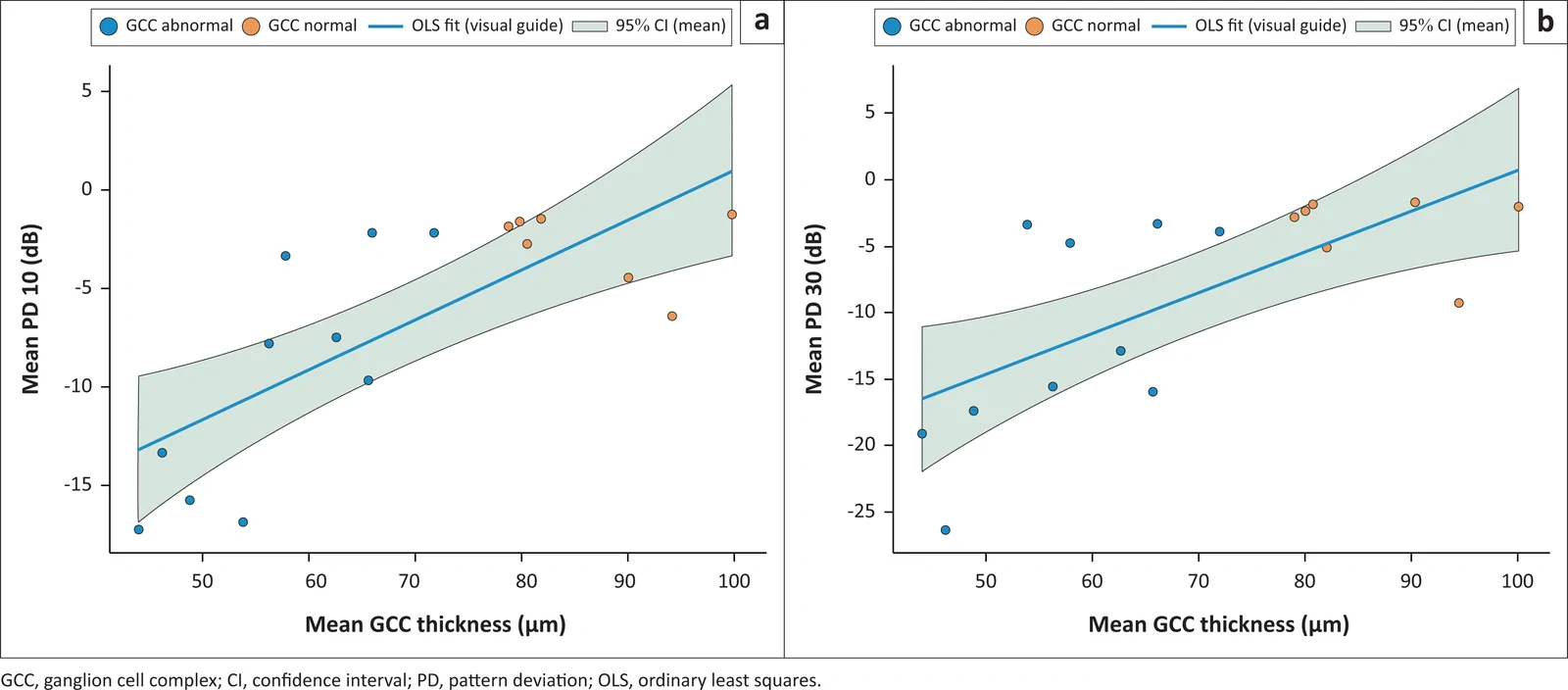
(a) Ganglion cell complex thickness versus pattern deviation 10-2 (eye-level), and (b) ganglion cell complex thickness versus pattern deviation 30-2 (eye-level).

A consistent relationship between structure and function was observed, with increased GCC thickness correlating with less negative PD values. At the eye level (*n* = 17), the mean GCC thickness demonstrated a significant association with the mean PD on 10-2 testing (*p* < 0.001) and 30-2 testing (*p* = 0.001) (Figure 2).

### Quadrant-level summary and mean pattern deviation plots

Quadrant-level summaries across included eyes (68 quadrant measurements) are presented in Table 3. On 10-2 testing, the most negative mean PD values occurred in quadrants 3 and 4 (VF inferior-temporal/GCC superior-nasal and VF superior-temporal/GCC inferior-nasal, respectively), whereas on 30-2 testing, the most negative mean PD values were observed in quadrant 4 (VF superior-temporal/GCC inferior-nasal) (see Table 3). Quadrant numbering is defined in the Methods according to the VF–GCC sector mapping (see Table 1 and Figure 1).

**TABLE 3:** Quadrant-level descriptive summary.

Quadrant	*n*	GCC		PD 10-2		PD 30-2	
		Mean	s.d.	Mean	s.d.	Mean	s.d.
1	17	70.5	19.8	−3.92	5.97	−7.07	7.79
2	17	75.6	16.3	−0.89	1.02	−4.52	4.93
3	17	65.8	18.8	−11.30	11.10	−10.56	12.65
4	17	65.1	20.1	−11.24	10.61	−12.49	10.15

Note: Quadrants are labelled according to the VF–GCC sector mapping in Table 1 and illustrated in Figure 1.

GCC, ganglion cell complex; PD, pattern deviation; s.d., standard deviation; *n*, number; VF, visual field.

**FIGURE 3:**
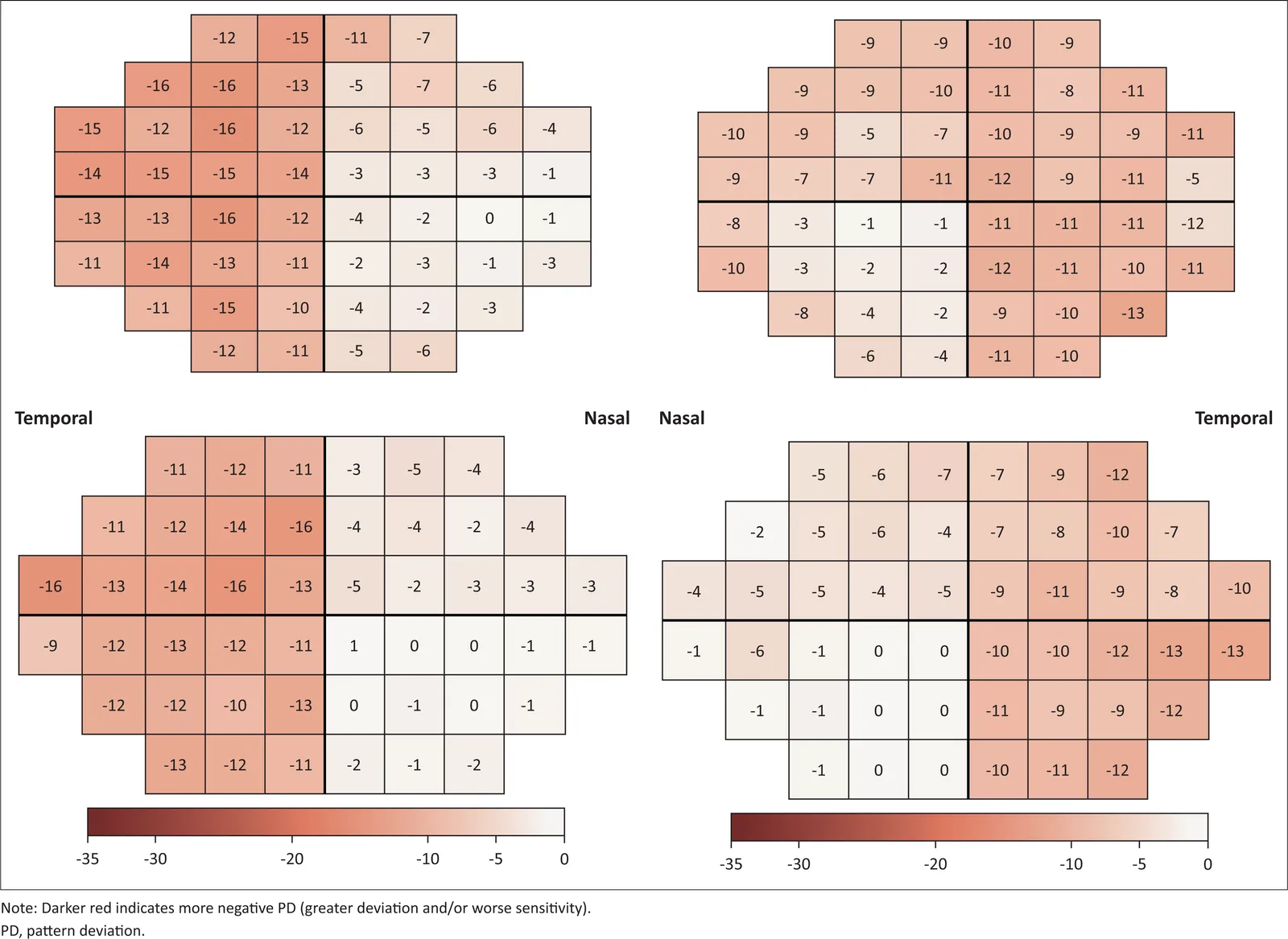
Mean pattern deviation number plots for Humphrey 30-2 (top row) and 10-2 (bottom row) testing, shown separately for right and left eyes.

Mean Humphrey-style PD number plots were generated by averaging pointwise PD values across the included eyes, presented separately for right and left eyes, and for both the 30-2 and 10-2 strategies (Figure 3). In the 30-2 testing, the mean PD plots revealed a broader field depression, with a clustering of more negative values in the temporal VF than in the nasal VF (Figure 3). During 10-2 testing, the mean PD number plots exhibited more localised central depression, again characterised by a predominance of negative values in the temporal VF relative to the nasal VF (Figure 3). The left eye 10-2 plot is based on *n* = 8 eyes, whereas the remaining mean plots are based on *n* = 9 eyes.

### Quadrant-level structure–function correlations (Ganglion cell complex vs visual field pattern deviation)

The structure–function correlations at the quadrant-level were evaluated in a cohort of 17 eyes, resulting in 17 paired observations per quadrant. Spearman correlation analyses revealed an overall positive association between GCC thickness and VF PD, indicating that increased GCC thickness is associated with less negative (i.e. more positive) PD values. During 10-2 testing, correlations were weak and not statistically significant in Quadrants 1 and 2 (VF inferior-nasal/GCC superior-temporal and VF superior-nasal/GCC inferior-temporal, respectively) (Quadrant 1: *p* = 0.442; Quadrant 2: *p* = 0.464), but they became more pronounced in the temporal VF quadrants. A robust correlation was observed in Quadrant 3 (VF inferior-temporal/GCC superior-nasal) (*p* = 0.004) and a moderate correlation in Quadrant 4 (VF superior-temporal/GCC inferior-nasal) (*p* = 0.018). In the 30-2 testing, the strongest correlations were observed in Quadrants 1 (VF inferior-nasal/GCC superior-temporal) (*p* = 0.0006) and 2 (VF superior-nasal/GCC inferior-temporal) (*p* = 0.006), with a moderate association noted in Quadrant 4 (VF superior-temporal/GCC inferior-nasal) (*p* = 0.021). Quadrant 3 (VF inferior-temporal/GCC superior-nasal) demonstrated a moderate correlation that did not reach statistical significance (*p* = 0.075). Collectively, these results suggest quadrant-dependent structure–function relationships, with the most consistent correlations observed for 30-2 PD in Quadrants 1, 2 and 4, and for 10-2 PD in Quadrants 3 and 4. The corresponding quadrant-level correlation plots are provided in the Online Appendix 1 (Figure 1-OA1–Figure 8-OA1).

### Agreement between binary classifications

The agreement was moderate between GCC and VF 10-2 (κ = 0.469; observed agreement 76.5%) and slight between GCC and VF 30-2 (κ = 0.164; observed agreement 64.7%). Discrepancies were primarily attributable to eyes classified as VF abnormal despite exhibiting normal GCC (10-2: 4 eyes; 30-2: 6 eyes) (Table 4).

**TABLE 4:** Agreement between binary classifications (eye-level; *N* = 17 eyes).

Comparison	Observed agreement		Cohen’s κ
	*n*	%	
GCC vs VF 10-2	13/17	76.5	0.469
GCC vs VF 30-2	11/17	64.7	0.164

GCC, ganglion cell complex; κ, Cohen’s kappa; VF, visual field.

### Visual acuity and visual fields outcomes

Visual acuity ranged from 6/4.5 to 6/24, with 13/17 eyes (76.5%) achieving ≥ 6/12. Using WHO-aligned logMAR categories, 13/17 eyes (76.5%) exhibited no impairment (logMAR ≤ 0.30), 2/17 (11.8%) demonstrated mild impairment (logMAR > 0.30 to 0.48), and 2/17 (11.8%) exhibited moderate impairment (logMAR > 0.48 to 1.00). Visual acuity category was not correlated with VF 10-2 abnormality (*p* = 0.541) or VF 30-2 abnormality (*p* = 1.000). A higher logMAR value, indicating poorer VA, was associated with more negative PD on both strategies (10-2: *p* = 0.015; 30-2: *p* = 0.011).

### Analytic note

All outcomes were analysed at the eye level. Most participants contributed both eyes (*n* = 7/10 participants; 14/17 eyes), while three participants contributed a single eye (3/10 participants; 3/17 eyes). As some participants contributed both eyes, findings should be interpreted with awareness of potential within-participant correlation.

### Longitudinal outcomes

Pre- and post-intervention time points were not consistently captured; therefore, the results reflect a cross-sectional assessment of structure–function at the time of testing.

## Discussion

In this cohort of patients with suprasellar tumours, VF abnormalities were prevalent across both strategies, with the 30-2 classification identifying more eyes as abnormal than the 10-2 classification. Abnormalities in the OCT macular GCC were less frequent than VF abnormalities. The agreement between normal and abnormal results was moderate when comparing GCC status on OCT to VF 10-2 status and slight when comparing GCC status on OCT to VF 30-2 status. Conversely, continuous measures revealed a consistent structure–function relationship, with a thinner GCC correlating with more negative PD in both VF strategies. Collectively, these findings imply that GCC thickness reflects functional loss on a graded continuum, whereas binary ‘normal or abnormal’ labels may fail to detect milder or early functional deficits.

The substantial burden and heterogeneity of VF loss are consistent with reports indicating that the ‘classic’ bitemporal hemianopia is not universally observed in pituitary-region disease. Additionally, VF patterns vary depending on lesion anatomy and its relationship to the anterior visual pathway.^3,4^ Contemporary reviews similarly emphasise the spectrum of VF patterns that may manifest with chiasmal compression, thereby underscoring the importance of implementing formal functional testing instead of relying on stereotyped patterns.^14^

Our findings endorse the utilisation of macular GCC thickness as a structural correlate of compressive optic neuropathy. Previous studies have demonstrated loss of the GCC in cases of chiasmal compression and have proposed that macular GCC asymmetry indicates chiasmal involvement.^8,9^ Structural alterations may be detectable in select patients prior to overt changes in functional classification, including reports of GCC or retinal nerve fibre layer thinning in macroadenomas without explicit chiasmal compression, as well as studies indicating that OCT analysis of the GCC can identify early chiasmal compression.^7,10^ The moderate concordance observed between dichotomised OCT and VF classifications within our cohort appears plausible and may reflect limitations inherent in applying normative cut-offs to continuous biological measures.^11^ Frameworks that compare structural and functional measures similarly highlight that dichotomising continuous parameters can diminish sensitivity near cut-points and obscure graded relationships.^11^ Clinically, discordant cases, particularly eyes classified as VF abnormal despite a ‘normal’ GCC indication, may represent early functional deterioration near threshold values, measurement variability or defects extending beyond the macular region. These observations further reinforce that OCT should be interpreted in conjunction with clinical and perimetric assessments rather than serving as a gatekeeper test. In cases of compressive chiasmopathy, significant associations between OCT parameters and VF loss have been demonstrated, including correlations between retinal nerve fibre layer thickness and VF sensitivity.^12,15^ Reviews evaluating OCT in compressive lesions of the anterior visual pathway also support the notion that GCC and retinal nerve fibre layer thinning serve as clinically useful biomarkers that correlate with functional impairment and can facilitate assessment and follow-up.^16^

Strategic considerations warrant attention when relating VF findings to macular structure. In our cohort, 30-2 classified more eyes as abnormal than 10-2, including two eyes that were normal on 10-2 but abnormal on 30-2. As 10-2 provides denser sampling of the central VF, it may better reflect macular GCC integrity in some patients, whereas broader grids may detect defects extending beyond the central field. These findings support strategy-aware interpretation and consistency of the chosen programme during follow-up. When monitoring is constrained by clinic time or test reliability, these results support a pragmatic approach: interpret GCC thickness as a continuous measure and use the VF strategy that best matches the clinical question (central function versus wider-field involvement), maintaining the same programme for follow-up where possible. In the South African public-sector context, where delayed presentation and high clinic volumes can limit repeatable, reliable perimetry, an objective OCT–derived measure, such as macular GCC thickness, may be a useful adjunct for baseline staging and follow-up planning, while recognising that functional testing remains essential.

Although this study was cross-sectional and did not assess postoperative recovery, the observed structure–function relationship remains clinically relevant. Prior research has established a link between tumour burden and visual outcomes, and measures of the macular ganglion cell layer have demonstrated prognostic significance in parasellar tumours.^13,17,18^ Optical coherence tomography may therefore play a role in staging severity and guiding follow-up planning in conjunction with VF testing.

This study has certain limitations. The sample size was modest and was drawn from a tertiary referral clinic, which may limit the generalisability of the findings. Analyses were performed at the eye level, and including both eyes from some participants could introduce within-participant correlation. Additionally, eyes with very poor acuity or consistently unreliable VF tests were excluded, potentially resulting in underrepresentation of patients with advanced or challenging-to-test disease. Radiological grading of chiasmal compression was not incorporated, thereby limiting the capacity to correlate anatomical severity. Larger, prospective studies that include radiological severity metrics and longitudinal assessments are necessary, ideally employing analytical methods that account for within-participant correlation and exploring whether integrated OCT and VF pathways enhance routine monitoring. Future research should also investigate how different VF testing strategies perform across varying radiological severity strata and determine whether central testing offers additional value over broader grids in predicting functional outcomes.

## Conclusion

Visual field abnormalities were frequently observed in patients with suprasellar tumours during both 10-2 and 30-2 testing. Conversely, abnormalities in the macular GCC assessed by OCT were less prevalent. The concordance between binary classifications of the GCC and VF was moderate for 10-2 and only slight for 30-2. However, continuous measurements of GCC thickness demonstrated a significant correlation with the VF PD across both testing strategies. These findings endorse the combined use of structural and functional assessments, while urging cautious interpretation of dichotomised OCT classifications and a strategy-aware approach to VF analysis.
